# LncRNA CD27-AS1 promotes acute myeloid leukemia progression through the miR-224-5p/PBX3 signaling circuit

**DOI:** 10.1038/s41419-021-03767-9

**Published:** 2021-05-18

**Authors:** Yanling Tao, Jingjing Zhang, Lulu Chen, Xin Liu, Mingkang Yao, Hao Zhang

**Affiliations:** 1 Department of Pediatric Hematology, Affiliated Hospital of Jining Medical University, Jining, Shandong Province China; 2 Department of Hematology, Affiliated Hospital of Jining Medical University, Jining, Shandong Province China; 3 Institute of Blood and Marrow Transplantation, Jining Medical University, Jining, Shandong Province China; 4 Graduate School, Department of Clinical Medicine, Jining Medical University, Jining, Shandong Province China

**Keywords:** Growth factor signalling, Oncogenesis

## Abstract

Acute myeloid leukemia (AML) is a hematological malignancy with a low cure rate, especially in the elderly. Previous studies have shown that long non-coding RNA (lncRNA) may be an important factor in the pathogenesis of hematological malignancies, including acute myeloid leukemia (AML). However, the biological roles and clinical significances of most lncRNAs in AML are not fully understood. LncRNA CD27 Antisense RNA 1 (CD27-AS1), as a member of lncRNA family, has rare reports on its function. In present study, we found that the expression of CD27-AS1 examined by quantitative real-time PCR was markedly increased in the AML patients (*N* = 40) compared with healthy volunteers (*N* = 40). The overall survival time was significantly shorter in patients with higher CD27-AS1 expression than that in patients with lower CD27-AS1 (*P* < 0.01). Furthermore, downregulation of CD27-AS1 in AML cells suppressed proliferative ability, arrested cell cycle in G0/G1 phase, and induced apoptosis. However, CD27-AS1 overexpression further enhanced the malignant phenotype of AML cells. Additionally, CD27-AS1 was proved to increase PBX3 expression through sponging miR-224-5p. CD27-AS1 knockdown blocked the MAPK signaling through PBX3 silencing and further inhibited the cell growth of AML cells. Taken together, we demonstrate that CD27-AS1 may be a potential prognostic biomarker of AML, and our finding also provides a new insight for non-coding RNA-based therapeutic intervention of AML.

## Introduction

Acute myeloid leukemia (AML) is a hematological malignancy derived from abnormally differentiated clonal myeloid cells^1,2^. AML occurs especially in the elderly population^2^. The cure rate of AML patients under 60 years old is 20–35% higher than that of patients over 60 years old^1^. Combined with the heterogeneity of AML cytogenetics, the therapeutic methods have become more thoughtful and specific^3^. Although the development of novel agents and improved treatment strategies have improved the prognosis and survival rate of AML patients, relapse and drug resistance after treatment are still the main problems affecting the cure of AML patients^2,4^. Therefore, exploring new diagnostic molecules or therapeutic targets is of great significance for the treatment of AML.

Long non-coding RNAs (lncRNAs), with a length longer than 200 nucleotides, are involved in the regulation of various physiological and pathological processes, including cancers^5^. LncRNA CD27 Antisense RNA 1 (CD27-AS1), as a member of lncRNA family, has rare reports on its function. Ma et al. showed that CD27-AS1 expression was significantly upregulated in melanoma, and knockdown of CD27-AS1 suppressed the malignant phenotype of melanoma cells^6^. However, the expression, function and molecular mechanism of CD27-AS1 in AML are completely unclear. According to the integrated analysis from the Cancer Genome Atlas (TCGA) and the Genotype-Tissue Expression (GTEx) databases, we found that the expression of CD27-AS1 in AML patient samples was significantly increased and negatively correlated with the prognosis of AML patients. We hypothesized that CD27-AS1 may exert important roles in the occurrence and development of AML.

A microRNA (miRNA) mainly regulates gene expression by silencing or degrading its target mRNA molecules to further affect various cellular processes, including tumor progression and metastasis^7^. It has been reported that downregulation of miR-224-5p in chronic myeloid leukemia cells was related to cell survival and chemoresistance^8^. LncRNAs can sponge miRNAs to affect downstream gene expression and function indirectly^9^. It was predicted that CD27-AS1 may be a potential target of miR-224-5p, and miR-224-5p may also bind to PBX Homeobox 3 (PBX3) from the online bioinformatics tools of Starbase and TargetScan. PBX3 was significantly upregulated in AML samples, and its expression was negatively correlated with AML patient prognosis^10^. Additionally, PBX3 could activate the mitogen-activated protein kinases (MAPK) signaling pathway to regulate cellular activities in several cancers^11–13^. Therefore, those findings suggest a miR-224-5p/PBX3/MAPK signaling pathway may be the downstream of CD27-AS1 to regulate AML progression. In this study, we aimed to investigate the expression and function of CD27-AS1 in AML and study the possible underlying mechanism involved in it.

## Materials and methods

### Clinical specimens and CD34+ cell isolation

The bone marrow samples of 40 AML patients (16 males and 24 females, median age 43, range 4–79) and 40 healthy donors (15 males and 25 females, median age 28, range 19–39) were obtained between January 2018 and June 2020. These samples were collected from the Affiliated Hospital of Jining Medical University according to the guidelines of the Declaration of Helsinki. All patients and healthy volunteers had signed informed consent before tissue collection. All experiments were approved by the Ethics Committee of the Jining Medical University.

Bone marrow mononuclear cells (BMNCs) were separated from bone marrow samples. The bone marrow samples were diluted with PBS in the same volume, and the diluted bone marrow was spread to the upper part of the separation liquid. After centrifugation, the buffy coat containing the mononuclear cells was collected. For CD34+ cell sorting, the mononuclear cells were then diluted with PBS and stained with trypan blue to count and the cell density was adjusted to 1 × 10^6^ cells/ml. After centrifugation, the cells were collected and incubated with Anti-Human CD34 PE staining buffer (5 μl) at 4 °C in the dark for 10 min. The cells were collected after centrifugation and resuspended with a staining buffer (500 μl), followed by flow cytometry for CD34+ cell sorting. As previously described^14^, recombinant human TPO (hTPO) (10 ng/ml, Sino Biological Inc., Beijing, China) was used to induce CD34+ cell proliferation.

### Cell culture

The human AML cell lines HL-60, Kasumi-1, and KG-1 were purchased from Procell (Wuhan, China). HL-60 and KG-1 cells were incubated in Iscove’s Modified Dulbecco Medium (IMDM, Procell) supplemented with 20% fetal bovine serum (FBS, HyClone, Utah, USA) and Kasumi-1 cells were cultured in Roswell Park Memorial Institute (RPMI)-1640 medium (Procell) supplemented with 20% FBS (HyClone). All the media were placed in an incubator at 37 °C and 5% CO_2_.

### Lentivirus construction and infection

The recombinant lentivirus plasmid pFUW (#14882, Addgene, Cambridge, USA) containing full length of CD27-AS1 (LV-CD27-AS1) between the BamHI and HpaI sites was constructed. Lentivirus plasmid Tet-pLKO-puro (#21915, Addgene) was used to achieve CD27-AS1 knockdown by inserting specific short hairpin RNAs (shRNAs) of CD27-AS1 into the region between the AgeI and EcoRI restriction sites: LV-CD27-AS1-Sh1 (target sequence: 5′-GCCTGTGTTCTGTCTCTTA-3′) and LV-CD27-AS1-Sh2 (target sequence: 5′-GCAGAAGGAGATCCGATG-3′). Pre-mir-224 (5′-GGGCTTTCAAGTCACTAGTGGTTCCGTTTAGTAGATGATTGTGCATTGTTTCAAAATGGTGCCCTAGTGACTACAAAGCCC-3′) and miR-224-5p sponge (5′-CTAAACGGAACCACTAGTGACTTGAcgatCTAAACGGAACCACTAGTGACTTGAtcacCTAAACGGAACCACTAGTGACTTGAtttttt-3′) were also ligated into the Tet-pLKO-puro vector to attain miR-224-5p overexpression and knockdown, respectively. Lentivirus plasmid pLVX-IRES-puro overexpressing PBX3 (LV-PBX3) was purchased from Fenghbio (#BR025, Changsha, China). The viral titer for all the lentivirus plasmids was 10^8^ TU/ml. AML cells were infected with relevant lentiviral vectors at a multiplicity of infection (MOI) of 10. Culture medium containing the lentivirus plasmids was replaced with fresh IMDM containing 20% FBS 24 h after incubation. Following functional analysis of the cells were carried out 72 h after infection.

### Quantitative real-time PCR (qRT-PCR)

Total RNA was extracted using RNA simple Total RNA Kit (Tiangen, Beijing, China). Reverse transcription reaction was performed through TIANScript M-MLV Reverse Transcriptase (Tiangen), combined with RNase inhibitor (Tiangen), dNTP, oligo (dT)^15^ or RT primer. The RT primer (for miR-224-5p) was: 5′-GTTGGCTCTGGTGCAGGGTCCGAGGTATTCGCACCAGAGCCAACCTAAAC-3′. Acquired cDNA was mixed with specific primers, SYBR Green (Solarbio, Beijing, China), and Taq DNA mix (Tiangen) to undergo quantitative real-time PCR reaction using an ExicyclerTM 96 fluorescence meter (Bioneer, Daejeon, Korea). Relative expression levels were quantified through the 2^−^^ΔΔCT^ method. The primers were: has-miR-224-5p forward: TCAAGTCACTAGTGGTTCCGTTTAG, and reverse: GCAGGGTCCGAGGTATTC. U6 forward: GCTTCGGCAGCACATATACT, and reverse: GCAGGGTCCGAGGTATTC. CD27-AS1 forward: TGTGACCTGCTAATGAATG, and reverse: GCTTGGGAGACAGAGTGA. GAPDH forward: GACCTGACCTGCCGTCTAG, and reverse: AGGAGTGGGTGTCGCTGT. The CD27-AS1 expression was expressed as fold-change relative to GAPDH, and U6 served as an internal reference for miR-224-5p expression.

### Western blotting

Total protein samples were extracted by RIPA lysis buffer (Solarbio) supplemented with 1 mM phenylmethylsulfonyl fluoride (PMSF, Solarbio). Mitochondrial protein extraction kit (Nanjing Jiancheng Bioengineering Institute, Jiangsu, China) was used to extract mitochondrial protein samples from infected AML cells. Protein concentration was quantified by a BCA Protein Assay Kit (Solarbio) according to the manufacturer’s instruction. Certain amount of protein samples (10–20 μg, 20 μl) were loaded to undergo SDS-PAGE, which were then transferred to PVDF membranes (Millipore, USA). Membranes were blocked in 5% non-fat milk powder (Sangon Biotech, Shanghai, China) or 5% BSA (Biosharp, Hefei, China), followed by primary antibody incubation at 4 °C overnight. The primary antibodies were: anti-Ki67 (Affinity, AF0198, China), anti-PCNA (Proteintech, 10205-1-AP, China), anti-cyclinD1 (Proteintech, 26939-1-AP), anti-cyclinE (Proteintech, 11554-1-AP), anti-CDK2 (Proteintech, 10122-1-AP), anti-CDK4 (Proteintech, 11026-1-AP), anti-p-RB (Ser795) (Cell signaling technology, CST, #9301, USA), anti-P21 (Proteintech, 10355-1-AP), anti-P53 (Proteintech, 10442-1-AP), anti-BCL-2 (Proteintech, 12789-1-AP), anti-Bax (Proteintech, 50599-2-lg), anti-cleaved caspase-3 (CST, #14220), anti-cleaved caspase-9 (Proteintech, 10380-1-AP), anti-cleaved PARP (CST, #9532), anti-cytochrome c (Proteintech, 10993-1-AP), anti-P38 (Affinity, AF6456), anti-p-P38 (Thr180/Tyr182) (Affinity, AF4001), anti-c-Jun NH 2 -terminal kinase (JNK, Affinity, AF6318), anti-p-JNK (Thr183/Tyr185) (Affinity, AF3318), anti-p-C-raf (Ser338) (Affinity, AF3065), anti-p-MEK1/2 (Ser218/Ser222) (Affinity, AF8035), anti-extracellular signal-regulated kinase (ERK, Affinity, AF0155), anti-p-ERK (Thr202/Tyr204) (Affinity, AF1015), anti-PBX3 (Affinity, DF8080), anti-COX IV (Gene Tex, GTX49132, USA), anti-GAPDH (Proteintech, 60004-1-Ig). Corresponding secondary antibodies goat anti-rabbit IgG-HRP (Solarbio, SE134) and goat anti-mouse IgG-HRP (Solarbio, SE131) were used to incubate membranes at 37 °C for 1 h. Enhanced chemiluminescence (ECL, Solarbio) reagent was then added to the membranes and densitometric analysis of protein bands was performed using Gel-Pro-Analyzer (Media Cybernetics, USA). The results were expressed as fold-change relative to GAPDH.

### CCK-8 assay

A total of 5 × 10^3^ cells (HL-60 or KG-1) were plated on 96-well-plates per well, with 100 μl medium containing relevant lentivirus and/or 10 μM U0126, a MEK1/2 inhibitor (MCE, New Jersey, USA), for 72 h. Five replicates were set in each condition. CCK-8 reagent (Keygen Biotech, Nanjing, China) was then added, followed by further culturing for 2 h at 37 °C in 5% CO_2_. The value of optical density at 450 nm was assessed using a Biotek ELX800 absorbance microplate reader.

### Flow cytometry detection

Flow cytometry was mainly used to detect cell cycle progression and apoptosis. Cell cycle analysis kit (Beyotime, Shanghai, China) was purchased to evaluate cell cycle distribution based on propidium (PI) staining, while an Annexin V-FITC apoptosis detection kit (Dojindo Laboratories, Kumamoto, Japan) was used to evaluate cell apoptosis through double staining of PI and Annexin V-FITC.

### Methylcellulose clonogenic assay

Cells in each group were harvested and centrifuged to collect cell precipitation. After supernatant removal, complete medium was added to completely resuspend the cells, and the cells were counted. After that, cells were seeded in petri dishes (100 cells each dish) and cultured in the IMDM medium (Procell) containing 30% FBS and 0.9% methylcellulose. After placed in an incubator for 2 weeks at 37 °C with 5% CO_2_, clone formation in petri dishes was recorded.

### Hoechst staining

Hoechst staining kit (Beyotime) was used to evaluate cell apoptosis after infection according to the manufacturer’s instruction. Mounted cells were observed under a fluorescence microscope (IX53; Olympus, Tokyo, Japan) at 400× magnification.

### Dual-luciferase reporter assay

AML cells (HL-60 and KG-1) were used to perform the dual-luciferase reporter assay. Potential binding sequences between CD27-AS1 and miR-224-5p, and miR-224-5p and PBX3 were predicted using Starbase and TargetScan. Cells were co-transfected with the luciferase plasmid pmirGLO (#E133A, Promega, Beijing, China) containing predicted wildtype or mutant sequences of binding targets and miR-224-5p mimic (or mimic-NC) through transfection reagent Lipofectamine 2000 (Invitrogen, NY, USA) according to manufacturer’s instruction. Relative luciferase activities (three replicates for each) were evaluated through the ratio of firefly luciferase to renilla luciferase using Dual-Luciferase Reporter Gene Assay Kit (Keygen Biotech) in accordance with the manufacturer’s protocol.

### Statistical analysis

All data were presented as mean with standard deviation (SD). All experiments were repeated at least three times. GraphPad Prism 8 was used to analyze data in this work, and ordinary one-way ANOVA in combination with Tukey’s multiple comparison test was applied to analyze differences between groups among three or more groups. The expression correlation between CD27-AS1 and miR-224-5p was analyzed via Pearson correlation analysis. *P* < 0.05 was regarded to be statistically significant.

## Results

### Expression of CD27-AS1 and miR-224-5p in AML patients and cell lines

**Fig. 1:**
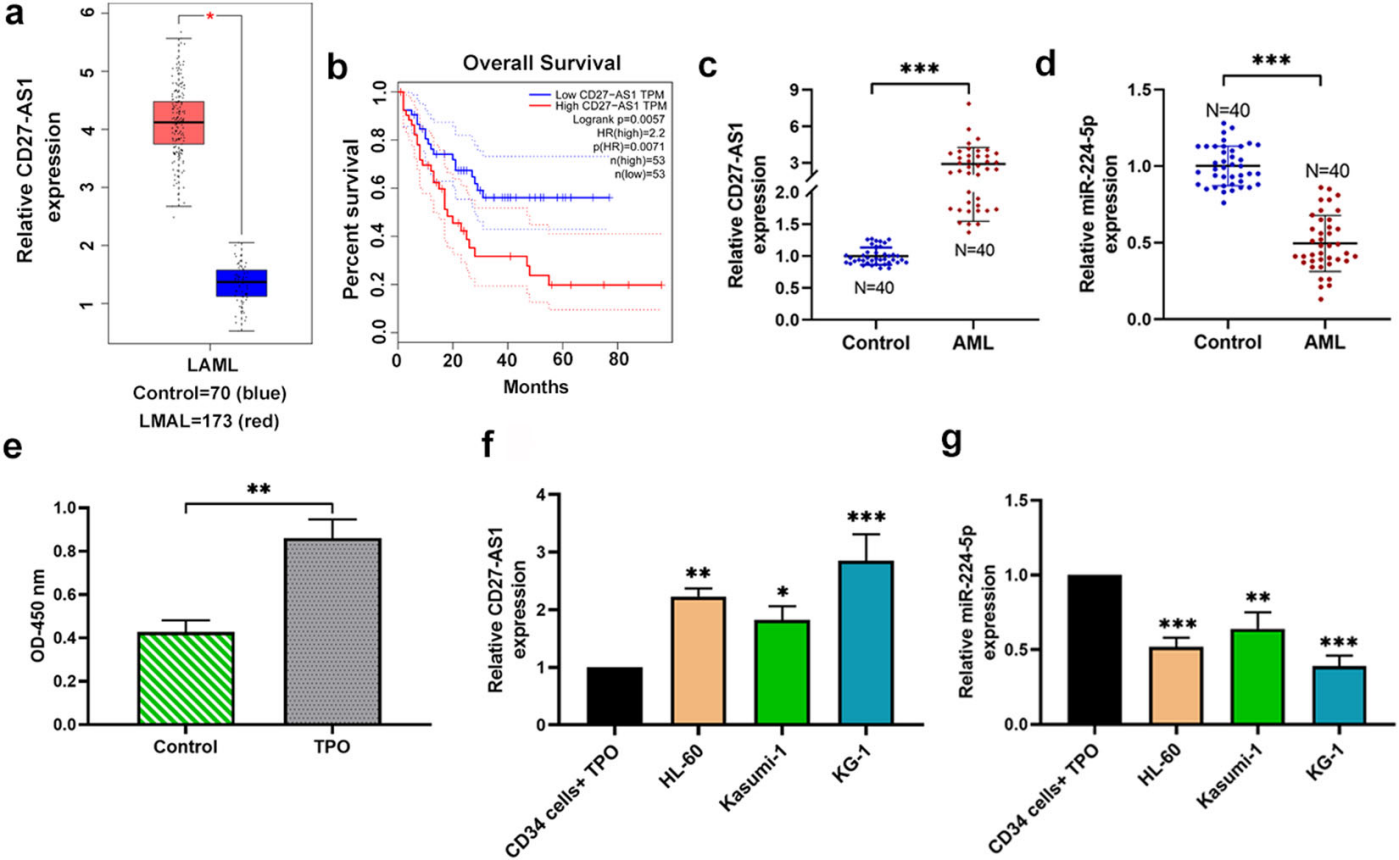
CD27-AS is overexpressed in AML samples and cell lines and is negatively corrected with the prognosis of AML patients. **a**, **b** Expression levels of CD27-AS1 in AML patient samples and the relationship between CD27-AS1 expression and prognosis of AML patients analyzed by the Cancer Genome Atlas and the Genotype-Tissue Expression databases. **c**, **d** Relative expression levels of CD27-AS1 and miR-224-5p in bone marrow mononuclear cells of AML patients (*N* = 40) and healthy volunteers (*N* = 40). **e** The viability of normal CD34+ cells treated with recombinant human TPO (hTPO) (10 ng/mL) was detected by CCK-8 assay. **f**, **g** Expression levels of CD27-AS1 and miR-224-5p in the TPO-treated CD34+ cells and AML cell lines (HL-60, Kasumi-1, KG-1) were detected using qRT-PCR. *N* = 3. Data were shown as means ± SD. *P < 0.05, **P < 0.01, and ***P < 0.001.

According to the integrated analysis from the Cancer Genome Atlas (TCGA) and the Genotype-Tissue Expression (GTEx) databases, we found that the expression of CD27-AS1 in AML patients was significantly increased, and negatively correlated with the prognosis of AML patients (Fig. 1a, b). In this study, we first detected the relative expression of CD27-AS1 and miR-224-5p in BMNCs of AML patients (*N* = 40) and healthy volunteers (*N* = 40) using qRT-PCR. As presented in Fig. 1c, d, CD27-AS1 expression was markedly increased in BMNCs of AML patients compared with healthy volunteers, whereas miR-224-5p expression was significantly decreased. Furthermore, we stimulated the proliferation of CD34+ cells with a growth factor TPO (Fig. 1e). Relative expression levels of CD27-AS1 and miR-224-5p in TPO-treated CD34+ cells and three AML cell lines (HL-60, Kasumi-1, and KG-1) were assessed. It was shown that compared to the TPO-treated CD34+ cells, CD27-AS1 was significantly upregulated in the AML cell lines, while the expression of miR-224-5p was downregulated (Fig. 1f, g).

### CD27-AS1 promotes cell proliferation in the AML cell lines

**Fig. 2:**
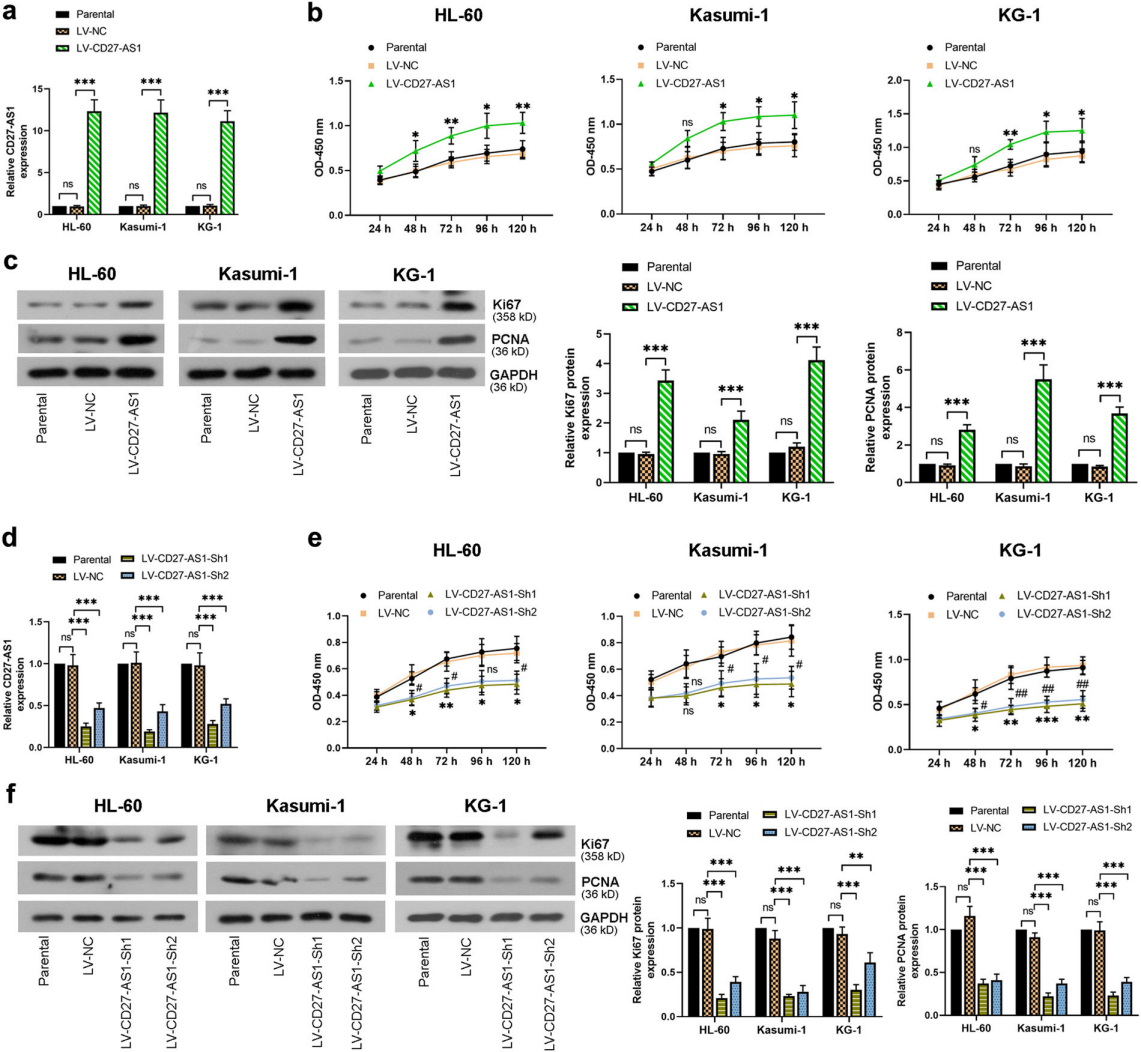
CD27-AS1 promotes cell proliferation in the AML cell lines. Three AML cells (HL-60, Kasumi-1, and KG-1) were infected with LV-CD27-AS1. **a** qRT-PCR was performed to check the relative expression of CD27-AS1 after infection among three cell lines. **b** CCK-8 assay showed cell proliferation after LV-CD27-AS1 infection in AML cells. *P < 0.05, **P < 0.01, ns = no significance versus the LV-NC. **c** Protein levels of Ki67 and PCNA in LV-CD27-AS1 infected AML cells were assessed using western blotting, and densitometry analysis was performed. Two LV-CD27-AS1-shRNAs were used to infect AML cells. **d** Relative expression of CD27-AS1 was assessed by qRT-PCR, after infection in HL-60, Kasumi-1, and KG-1 cells. **e** Cell proliferation was detected using CCK-8 assay. *P < 0.05, **P < 0.01, ***P < 0.001, ns = no significance in LV-CD27-AS1-Sh1 versus the LV-NC; ^#^*P* < 0.05, ^##^*P* < 0.01, ns = no significance in LV-CD27-AS1-Sh2 versus the LV-NC. **f** Western blotting was performed to check relative protein levels of Ki67 and PCNA after LV-CD27-AS1-shRNAs infection in three AML cell lines, with their quantification. *N* = 3. Data were shown as means ± SD. **P < 0.01, ***P < 0.001 and ns = no significance.

To study the effects of CD27-AS1 on cell proliferation, the three AML cell lines and normal CD34+ cells were infected with LV-CD27-AS1 and LV-CD27-AS1-Sh1/2. Both infections were efficient (Fig. 2a, d). CCK-8 assay showed that overexpression of CD27-AS1 enhanced cell proliferation over time in the AML cells and normal CD34+ cells, whereas knockdown of CD27-AS1 significantly inhibited cell proliferation (Fig. 2b, e and Supplementary Fig. 1a, b). Furthermore, protein levels of cell proliferative marker Ki67 and PCNA were evaluated by western blotting, showing that overexpression of CD27-AS1 remarkably increased the Ki67 and PCNA levels, but CD27-AS1 knockdown reduced these protein expression levels significantly (Fig. 2c, f). Therefore, the data suggested that CD27-AS1 could promote AML and normal CD34+ cell proliferation.

### Knockdown of CD27-AS1 arrests cell cycle progression and inhibits colony formation in the AML cell lines

**Fig. 3:**
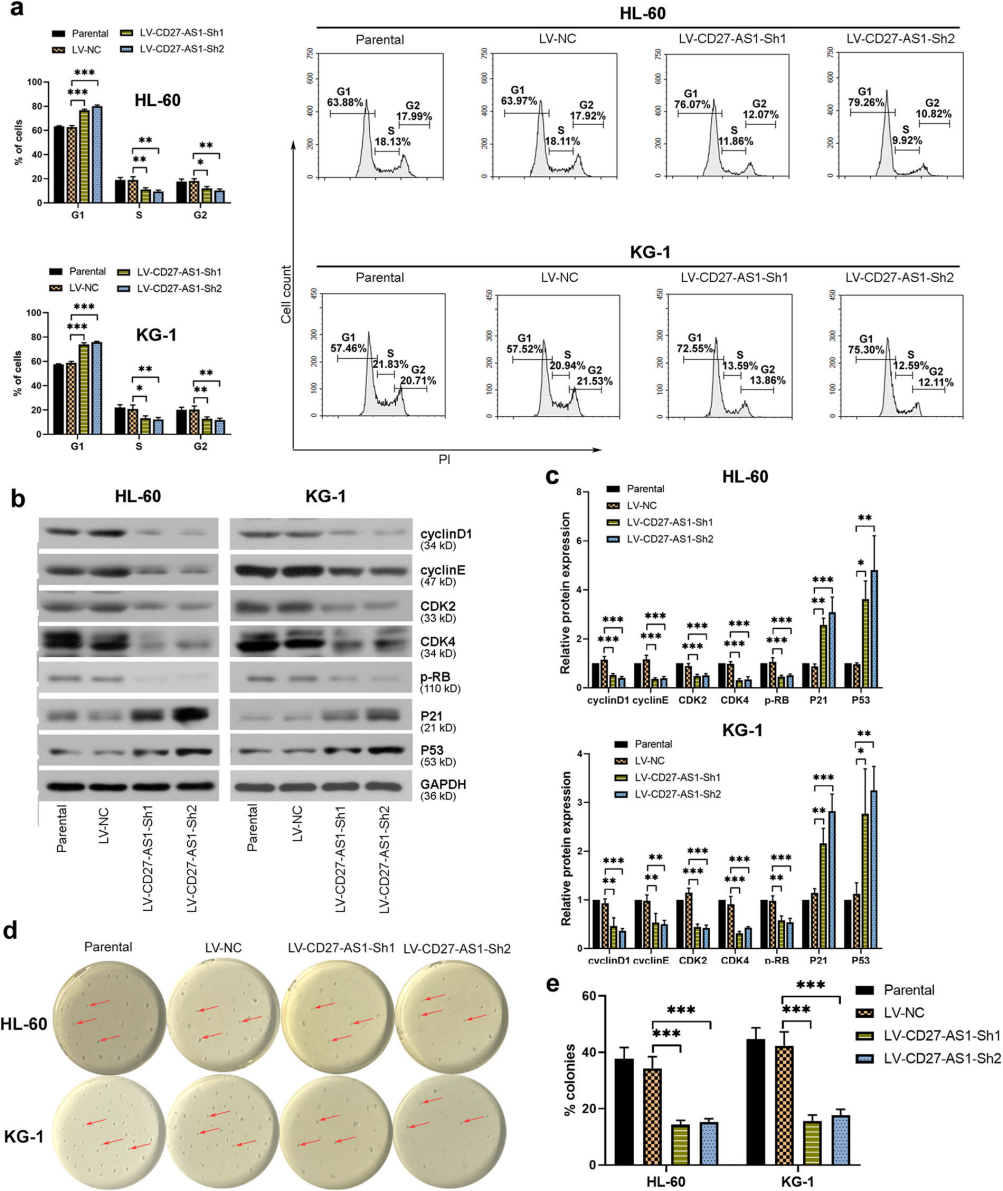
Knockdown of CD27-AS1 inhibits cell cycle progression and colony formation in the AML cell lines. HL-60 and KG-1 cells were infected with two LV-CD27-AS1-shRNAs. **a** Flow cytometry was performed to assess cell cycle distribution in infected AML cells. **b** Western blotting was used to check protein expression levels of cyclinD1, cyclinE, CDK2, CDK4, p-RB, P21, and P53 in the AML cells with CD27-AS1 knockdown. **c** Quantification of protein levels from western blotting was analyzed. **d**, **e** Colony formation of AML cells was detected by using methylcellulose clonogenic assay. *N* = 3. Data were shown as means ± SD. *P < 0.05, **P < 0.01, and ***P < 0.001. AML, acute myeloid leukemia.

AML cells HL-60 and KG-1 with higher expression of CD27-AS1 were used to infect with LV-CD27-AS1-shRNAs, to study the effects of CD27-AS1 on AML cell cycle distribution and colony growth. Results from flow cytometry showed that CD27-AS1 knockdown arrested more cells in the G1 phase, leading to significantly reduced cells in S/G2 phases (Fig. 3a). Further western blotting of cell cycle-related proteins also confirmed this finding, in which CD27-AS1 knockdown decreased protein levels of cyclinD1, cyclin E, CDK2, CDK4, and phosphorylated RB, but enhanced expression levels of P21 and P53 in both HL-60 and KG-1 cells (Fig. 3b, c). Moreover, methylcellulose clonogenic assay showed that the colony growth of HL-60 and KG-1 cells was markedly inhibited when CD27-AS1 was silenced (Fig. 3d, e). Collectively, knockdown of CD27-AS1 suppressed cell cycle progression and colony formation in the AML cells.

### Knockdown of CD27-AS1 enhances cell apoptosis in the AML cell lines

**Fig. 4:**
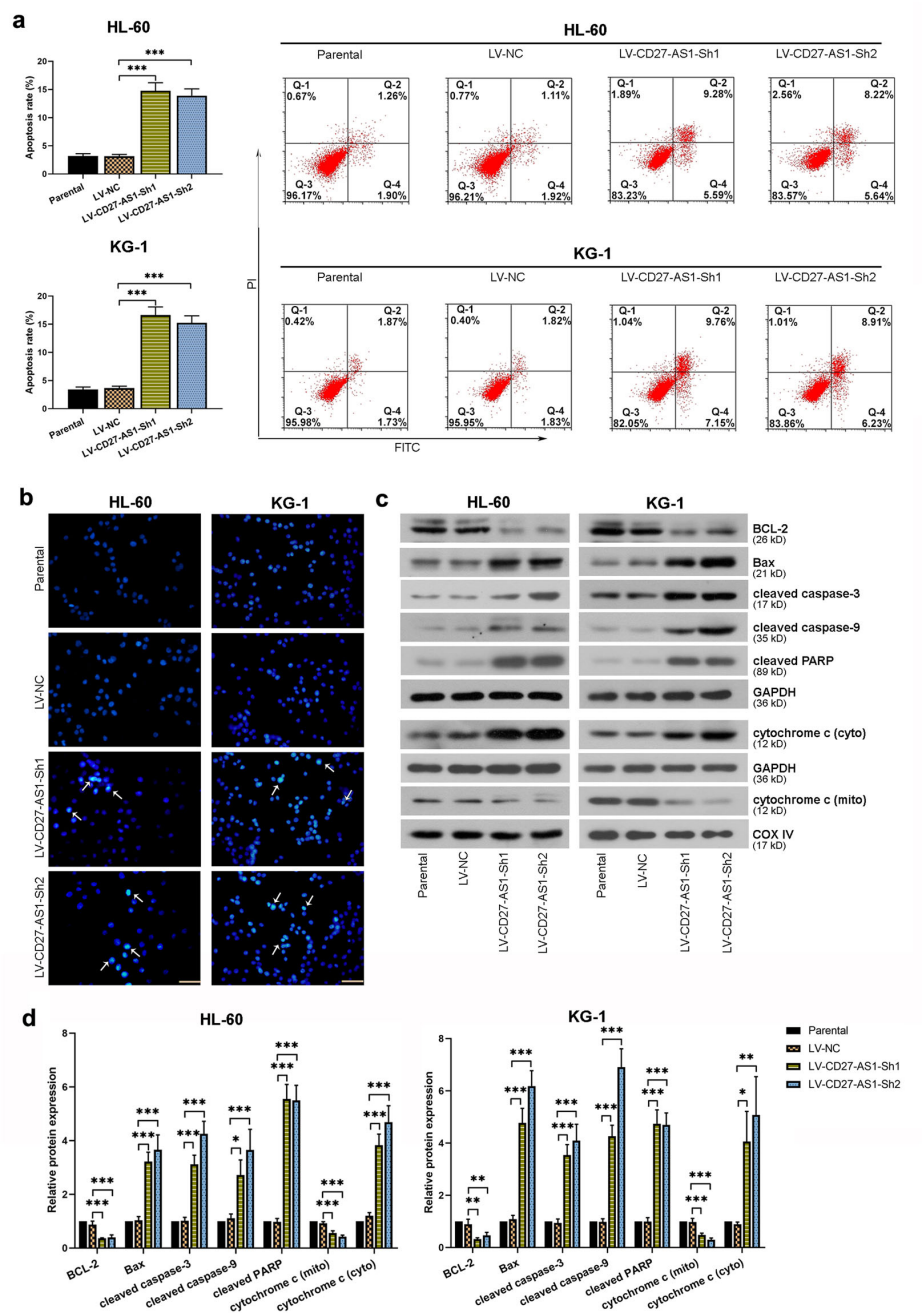
Knockdown of CD27-AS1 enhances cell apoptosis in the AML cell lines. **a** Cell apoptosis was evaluated using flow cytometry in HL-60 and KG-1 cells after LV-CD27-AS1-shRNA infection. **b** Hoechst staining was performed in two AML cells with CD27-AS1 knockdown. **c** Western blotting assay showed relative protein levels of BCL-2, Bax, cleaved caspase-3, cleaved caspase-9, cleaved PARP, and cytochrome c in cytoplasm and mitochondria in the AML cells after infection. **d** Quantitative analysis of above protein levels in the HL-60 and KG-1 cells. *N* = 3. Data were shown as means ± SD. *P < 0.05, **P < 0.01, and ***P < 0.001.

Effects of CD27-AS1 knockdown on AML cell apoptosis were evaluated subsequently. Both HL-60 and KG-1 cells infected with LV-CD27-AS1-shRNAs showed significantly increased cell apoptosis rate from flow cytometry results (Fig. 4a). Hoechst staining of infected cells was performed to detect the morphological alterations of cell apoptosis, which showed that CD27-AS1 knockdown led to obviously chromatin condensation in the AML cells (Fig. 4b). Similarly, some apoptosis-related protein levels were also checked by western blotting. The results showed that downregulation of CD27-AS1 could increase protein expression of Bax, cleaved caspase-3, cleaved caspase-9, cleaved PARP, and cytochrome c in the cytoplasm, but reduce BCL-2 and mitochondrial cytochrome c levels in the AML cells (Fig. 4c, d). Therefore, it was suggested that knockdown of CD27-AS1 enhanced AML cell apoptosis.

### CD27-AS1 mediates MAPK signaling pathway in the AML cell lines

**Fig. 5:**
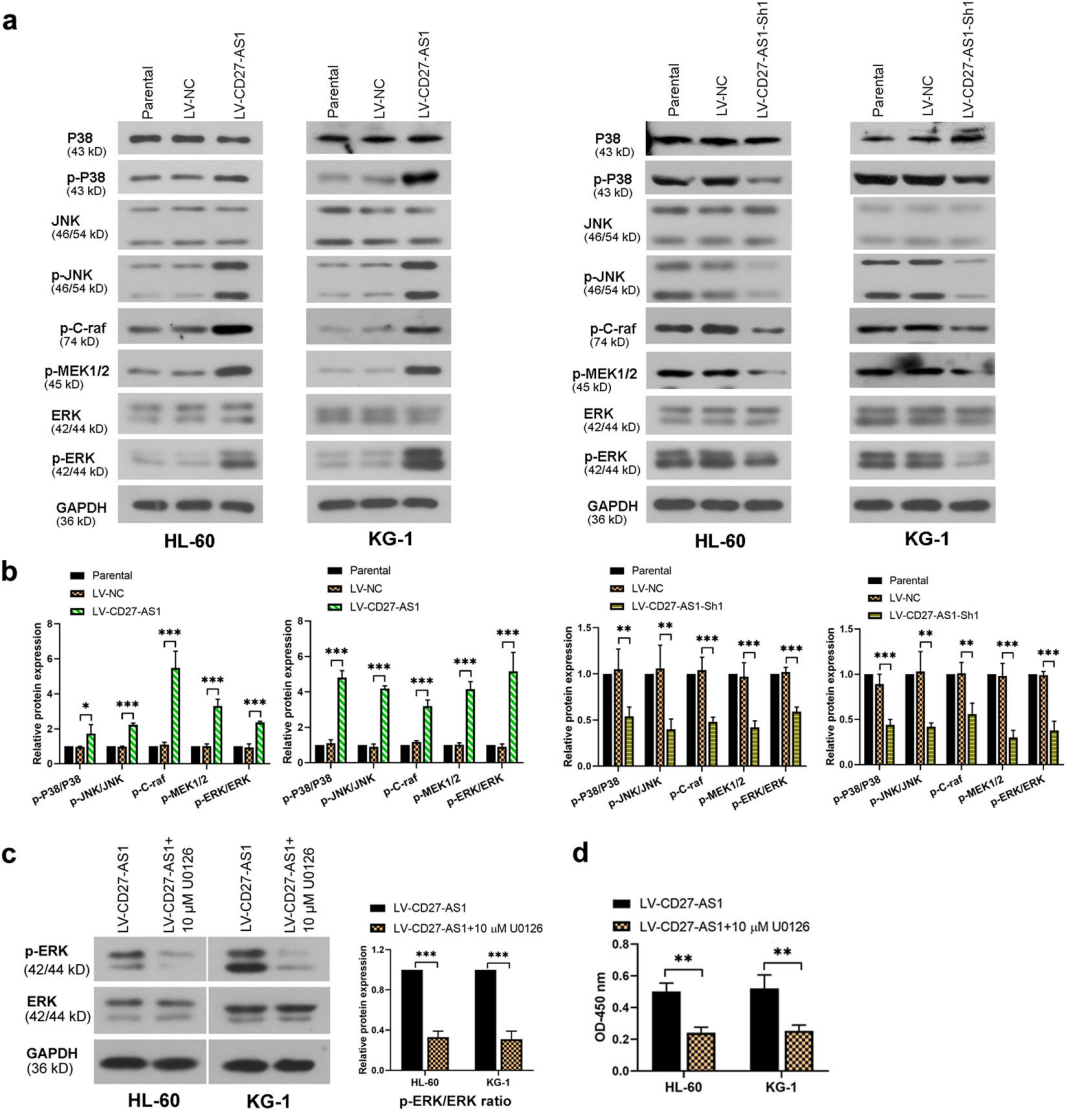
CD27-AS1 regulates MAPK signaling pathway in the AML cell lines. **a** HL-60 and KG-1 cells were infected with LV-CD27-AS1 and LV-CD27-AS1-Sh1. Protein levels of P38, p-P38, JNK, p-JNK, p-C-raf, p-MEK1/2, ERK, and p-ERK were detected using western blotting. **b** Densitometry analysis of protein levels in both cells was performed. **c** U0126, a MEK1/2 inhibitor, was used to treat the AML cells with CD27-AS1 upregulation. Relative protein levels of p-ERK and ERK were measured using western blotting. **d** CCK-8 assay was performed to check cell viability. *N* = 3. Data were shown as means ± SD. *P < 0.05, **P < 0.01, and ***P < 0.001.

To figure out whether MAPK signaling was involved in the regulation of CD27-AS1 in AML cells, three MAPKs and their phosphorylated forms were checked by western blotting. It was shown that the phosphorylated form of all three MAPKs (p-P38, p-JNK, and p-ERK) was enhanced in CD27-AS1 overexpressed AML cells (HL-60 and KG-1), while decreased in the AML cells with CD27-AS1 knockdown (Fig. 5a, b). Especially, we detected the upstream molecules of ERK signaling. Protein levels of p-C-raf and p-MEK1/2 were significantly increased when CD27-AS1 was upregulated, and knockdown of CD27-AS1 led to opposite effects in both HL-60 and KG-1 cells (Fig. 5a, b), indicating that CD27-AS1 activated MAPK signaling pathway in the AML cells. To verify this finding, U0126, a MEK1/2 inhibitor, was used to treat LV-CD27-AS1-infected AML cells. As shown in Fig. 5c, U0126 treatment significantly decreased the protein level of p-ERK in AML cells. Results from CCK-8 assay showed that U0126 treatment markedly decreased cell viability in CD27-AS1 overexpressed AML cells (Fig. 5d). Therefore, the results indicated that CD27-AS1 could mediate MAPK signaling in the AML cells.

### CD27-AS1 regulates AML cell activity by directly targeting miR-224-5p

**Fig. 6:**
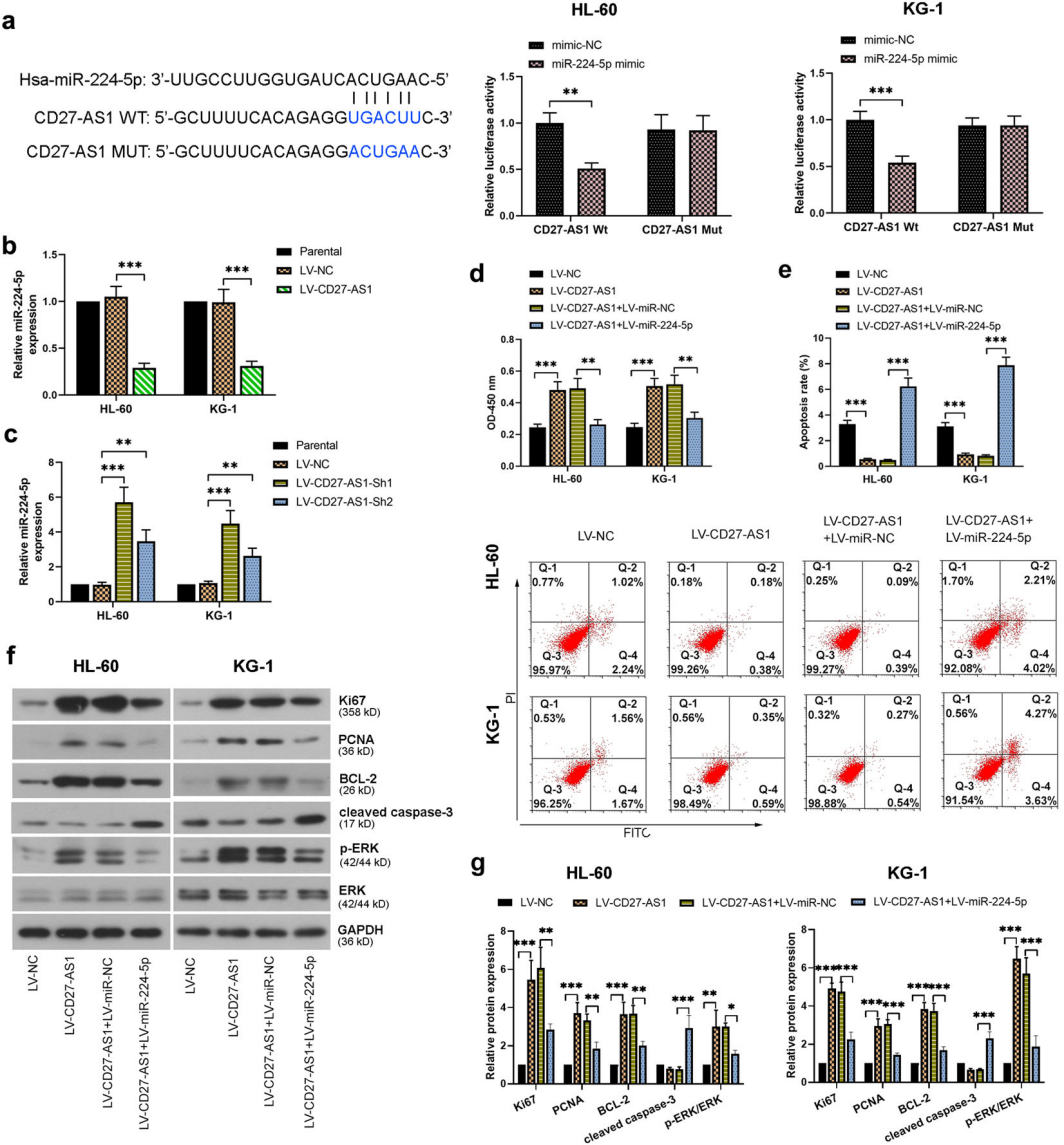
CD27-AS1 regulates AML cell activity by directly targeting miR-224-5p. **a** Dual luciferase reporter assay was carried out to verify the binding effects of miR-224-5p on CD27-AS1. Potential binding sequences between them were predicted from Starbase database. **b**, **c** Relative miR-224-5p expression was assessed in HL-60 and KG-1 cells with CD27-AS1 overexpression and CD27-AS1 knockdown, respectively, using qRT-PCR. Two AML cells were co-infected with the LV-CD27-AS1 and LV-miR-224-5p. **d** Cell viability and (**e**) cell apoptosis were then detected using CCK-8 assay and flow cytometry, respectively. **f** Western blotting was performed to check protein levels of Ki67, PCNA, BCL-2, cleaved caspase-3, ERK, and p-ERK in the AML cells after co-infection. **g** Quantification of above protein expression from western blotting. *N* = 3. Data were shown as means ± SD. *P < 0.05, **P < 0.01, and ***P < 0.001.

In contrast to CD27-AS1, the bioinformatics website (Oncolnc, http://www.oncolnc.org/) analysis showed that the expression of miR-224-5p was positively associated with the overall survival of AML patients (Supplementary Fig. 2b). Correlation analysis showed that the expression of miR-224-5p in BMNCs of AML patients (*N* = 40) was negatively correlated with CD27-AS1 (Supplementary Fig. 2a). To further investigate the correlation between CD27-AS1 and miR-224-5p in AML cells, dual-luciferase reporter assay was firstly performed according to the predicted binding sequence of miR-224-5p on CD27-AS1 (Fig. 6a). Both HL-60 and KG-1 cells showed lower luciferase activities when cells were co-transfected with miR-224-5p mimic and wildtype CD27-AS1 (Fig. 6a). Furthermore, CD27-AS1 overexpression caused decreased miR-224-5p expression (Fig. 6b), whereas CD27-AS1 knockdown significantly enhanced miR-224-5p expression in the AML cells (Fig. 6c). These data showed that CD27-AS1 targeted miR-224-5p and negatively regulated its expression. Subsequently, HL-60 and KG-1 cells were co-infected with LV-CD27-AS1 and LV-miR-224-5p, to study the effects of miR-224-5p on the regulation of CD27-AS1 in the AML cells. Three days after co-infection, cell viability and apoptosis were evaluated by CCK-8 and flow cytometry, respectively, which showed that further miR-224-5p introduction significantly reduced cell viability, but increased cell apoptosis in CD27-AS1-overexpressed/silenced AML cells (Fig. 6d, e and Supplementary Fig. 3a–c). Protein levels of typical indicators were also detected by western blotting, which showed that miR-224-5p introduction significantly reduced protein levels of Ki67, PCNA, BCL-2, and p-ERK/ERK, but dramatically increased cleaved caspase-3 levels in the AML cells overexpressing CD27-AS1 (Fig. 6f, g). The alterations of protein expression were consistent with the above cellular observations, suggesting that the regulation of CD27-AS1 in the AML cells was mediated by miR-224-5p through directly binding.

### MiR-224-5p binds to PBX3 to regulate AML cell activity

**Fig. 7:**
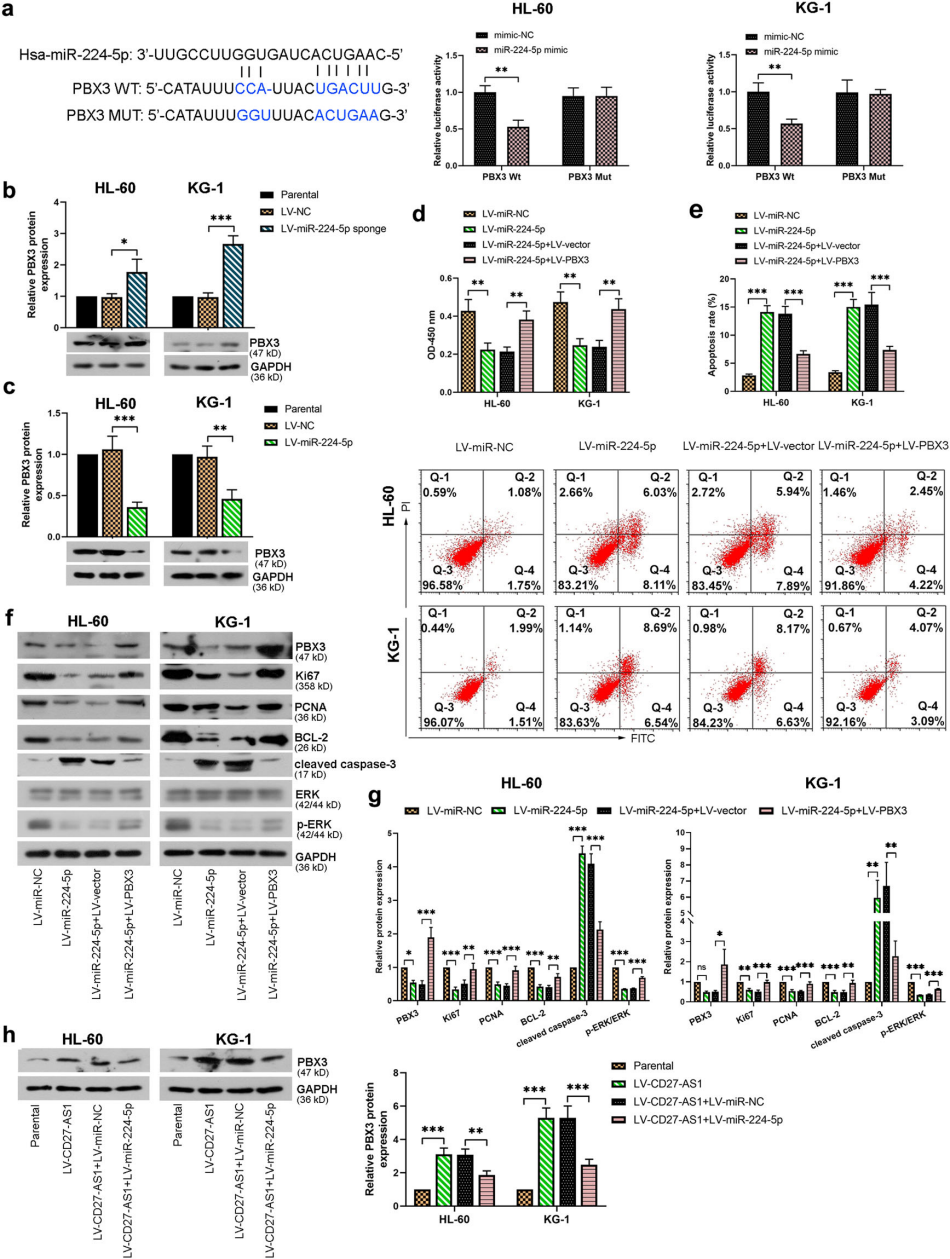
MiR-224-5p binds to PBX3 to regulate AML cell activity. **a** Binding sequences between miR-224-5p and PBX3 were shown, and their binding effects were evaluated using dual luciferase reporter assay. **b**, **c** Western blotting was used to show relative protein levels of PBX3 in HL-60 and KG-1 cells infected with LV-miR-224-5p sponge and LV-miR-224-5p. HL-60 and KG-1 were then co-infected with the LV-miR-224-5p and LV-PBX3. **d**, **e** CCK-8 and flow cytometry was performed to detect the cell viability and cell apoptosis, respectively. **f** Relative protein levels of PBX3, Ki67, PCNA, BCL-2, cleaved caspase-3, ERK, and p-ERK in two co-infected AML cells were evaluated using western blotting. **g** Quantified figures of above protein levels from western blotting. **h** PBX3 protein expression was assessed in HL-60 and KG-1 cells after co-infection of LV-CD27-AS1 and LV-miR-224-5p by western blotting, with their quantification. *N* = 3. Data were shown as means ± S.D. *P < 0.05, **P < 0.01, ***P < 0.001, and ns = no significance.

The effects of miR-224-5p on PBX3 were also evaluated in HL-60 and KG-1 cells. Dual-luciferase reporter assay showed that miR-224-5p also targeted PBX3 directly, as evidenced by reduced luciferase activity in the AML cells transfected with miR-224-5p mimic and wildtype PBX3 (Fig. 7a). Relative protein expression of PBX3 was increased in the AML cells with miR-224-5p knockdown (Fig. 7b), but reduced in the miR-224-5p overexpressed AML cells (Fig. 7c). Furthermore, the introduction of LV-miR-224-5p significantly decreased cell viability but elevated cell apoptosis rate in the AML cells, which were reversed by further PBX3 overexpression (Fig. 7d, e). Again, protein levels of PBX3, Ki67, PCNA, BCL-2, and p-ERK/ERK were reduced in the AML cells infected with LV-miR-224-5p, all of which could be reversed by following PBX3 overexpression, leading to increased protein expression levels (Fig. 7f, g). Besides, the changes of cleaved caspase-3 were opposite under the same condition (Fig. 7f, g). Notably, the protein levels of PBX3 were also assessed under co-infection of LV-CD27-AS1 and LV-miR-224-5p in HL-60 and KG-1 cells. It was shown that CD27-AS1 overexpression led to significantly increased PBX3 levels, which could be alleviated by further miR-224-5p introduction in the AML cells (Fig. 7h). Taken together, miR-224-5p targeted by CD27-AS1 regulated AML cell activity by targeting PBX3/MAPK signaling pathway.

## Discussion

LncRNAs have been found to be dysregulated in various cancer types, and aberrant expression allows them to modulate downstream gene expression and cell homeostasis abnormally, leading to malignant transformation^5^. In this study, we found that CD27-AS1 was highly expressed in AML patient samples and negatively correlated with the prognosis of AML patients, indicating that CD27-AS1 may serve an important role in the occurrence and development of AML. Previous studies have shown that the expression of lncRNA is affected by many factors, such as RNA binding proteins, transcription factors, DNA methylation, and histone modification^15^. It was previously reported that transcription factor SP1 was able to induce lncRNA expression through promoting gene transcription, including lncRNA PANDAR^16^, lncRNA AGAP2-AS1^17^, and lncRNA DANCR^18^. SP1 was overexpressed in AML samples, and exerted pro-tumor activity in leukemia^19,20^. Of note, bioinformatics website predicted that SP1 is capable of binding to the promoter of CD27-AS1 (http://jaspar.genereg.net/), and its expression is positively correlated with CD27-AS1 (http://gepia.cancer-pku.cn/). Moreover, Wang et al found that Suppressor of Ty Homologue-6 (SUPT6H) was upregulated in AML and regulated AML cell growth^21^. SUPT6H is a potential RNA binding protein that that is predicted to CD27-AS1 mRNA (https://rnact.crg.eu/). The evidence above suggests that CD27-AS1 expression may be regulated by transcription factors, such as SP1, or RNA binding proteins, such as SUPT6H.

The gain-of-function analysis showed that CD27-AS1 overexpression promoted cell proliferation in HL-60 and KG-1 cells. Additionally, CD27-AS1 overexpression also promoted normal CD34+ cell proliferation. Considering a basal expression of CD27-AS1 in normal CD34+ cells, it is not unpredictable that CD27-AS1 may also play a functional role in normal CD34+ cells. So far, CD27-AS1 has only been reported to be aberrantly expressed in several cancers, such as melanoma and cervical cancer^22,23^. Studies have shown that CD27-AS1 was upregulated in melanoma cell lines, and knockdown of it suppressed the growth and migration of melanoma cells^6^. The expression and effects of CD27-AS1 in melanoma development were similar with that in AML from current study. However, we know little about the specific roles of CD27-AS1 in other cancer types from published articles. Additionally, some other lncRNAs were also identified to participate in the AML progression and prognosis. Studies indicated that lncRNA urothelial carcinoma-associated 1 (UCA1) was upregulated in the AML cell lines and exerted oncogenic functions in AML^24,25^. IRAIN (insulin-like growth factor type I receptor-IGF1R antisense intragenic noncoding RNA) was downregulated in AML cells and patient samples, promoting intrachromosomal interaction of insulin-like growth factor receptor (IGF-1R) that can regulate AML cell growth^26^. Besides, lncRNAs like maternally expressed 3 non-protein-coding gene (MEG3), RUNX1 overlapping RNA (RUNXOR), nuclear paraspeckle assembly transcript 1 (NEAT1) were also dysregulated and exert specific functions in AML. However, their underlying molecular mechanism remains unclear^27^.

MAPK signaling is known to regulate various aspects of cellular activities, including proliferation, migration, differentiation, survival, and death^28^. As members of MAPKs, the ERK signaling is mainly activated by peptide growth factors, while the P38 and JNK signaling pathways are activated by a variety of stimuli, such as endoplasmic reticulum (ER) stress, oxidative stress, and inflammatory cytokines^28,29^. In our study, we detected all three MAPK signaling in the AML cells, and found that phosphorylated forms of all three MAPKs were enhanced significantly in the AML cells by CD27-AS1 overexpression, indicating that all three MAPKs were involved in the cell development through regulation of CD27-AS1. Activation of MAPK signaling can activate transcription factors and further increase cell proliferation and protect cells against apoptosis^30^. For instance, the ERK/MAPK pathway induced the activation of transcription factor AP-1 and further promoted the transcription of cyclin D1^31,32^.

The predicted binding effects between CD27-AS1 and miR-224-5p were also verified. It was studied that miR-224-5p was involved in the regulation of hsa_circ_0121582 on AML cell proliferation through the inhibition of Wnt/β-catenin signaling via targeting glycogen synthase kinase-3β^33^. Herein, we demonstrated that CD27-AS1 promoted AML progression through targeting miR-224-5p. Aside from CD27-AS1, miR-224-5p was indeed regulated by multiple lncRNAs, such as LncRNA MALAT1^34^ and NEAT1^35^ in other human diseases. MALAT1 promoted the malignant phenotype of AML cells^36^, whereas lncRNA NEAT1 inhibited the development of AML^37^. These above findings indicate the possibility that miR-224-5p is regulated by multiple lncRNAs in AML. Meanwhile, a single lncRNA is considered to sponge multiple miRNAs. Bioinformatics website predicts that CD27-AS1 may target miR-204-5p and miR-628 (Supplementary Fig. 2c, d). These two miRNAs were both negative regulators of AML development and inhibited the growth of AML cells^38,39^. The findings indicated that the CD27-AS1/miR-224-5p axis may be one of the ways that CD27-AS1 regulates the growth of AML cells. Additionally, various studies have linked AML cell differentiation to disruption of miRNAs, including let-7c^40^, miR-638^41^, and miR-128a^42^, in which let-7c and miR-638 overexpression promoted granulocytic differentiation of AML cells, while miR-128a overexpression inhibited macrophage- and granulocytic-like differentiation of AML cells. Whether CD27-AS1/miR-224-5p axis regulates the differentiation of AML cells is unknown, which needs further study in the future.

The miRNA recognition element on coding genes allows miRNA to directly target them and perform miRNA-mediated regulation^43^ Currently, the function and regulation mechanism of miR-224-5p in the development of AML remains unclear. However, in other pathological processes, miR-224-5p has been reported to target multiple genes, including AKT3^44^, Rac1^45^, and Rab10^46^. These three factors were able to promote the growth of AML cells^47–49^. The findings indicate that miR-224-5p may regulate the growth of AML cells by targeting multiple genes. In present study, we confirmed one downstream target of miR-224-5p, PBX3 in the AML cells. PBX3 was reversely correlated with the miR-224-5p expression, and also reversed the miR-224-5p-induced reduction of cell viability and increase of apoptosis in the AML cells, indicating that miR-224-5p regulate the growth of AML cells through targeting PBX3. Previous studies indicated that PBX3 was highly expressed in AML clinical samples, and AML mice with *PBX3* deletion had an extended survival time^10^. PBX3 was an essential cofactor of HOXA genes during leukemogenesis^50^, and the HOXA/PBX3 signature was also mediated by upstream miRNAs, including miR-181 and miR-335, in AML^51,52^. Except for the reported oncogenic role of PBX3 in AML, upregulated PBX3 has also been found in other cancers, such as gastric cancer, colorectal cancer, liver cancer, glioma, etc., that is related to the malignant transformation and poor prognosis^53^. The interaction of PBX3 and MAPK signaling was studied in several cancers. Specifically, PBX3 knockdown significantly reduced the increased phosphorylation level of Raf-1, p38, and ERK1/2 in glioma cells^54^. In this study, we also showed activated MAPK signaling after PBX3 upregulation in the AML cells. Previous studies reported that blocking PBX3/Meis Homeobox 1 (MEIS1) dimerization could inhibit cell proliferation and suppressed Tribbles Homolog 2 (TRIB2) expression^55^. TRIB2 is pseudokinase identified as an oncogene in AML^56^, and its deficiency resulted in impaired activation of MAPKs in AML cells^57^. In papillary thyroid carcinoma cells, PBX3 increased the expression of Kinase Insert Domain Receptor (KDR), a type III receptor tyrosine kinase^58^. Inhibition of KDR with pharmaceutical inhibitors^59,60^ or neutralizing antibody^59^ showed anti-AML effects−inhibited cell proliferation and induced cell apoptosis. Interestingly, blockade of KDR abrogated MAPK signaling transduction in AML cells^61^. The above literatures indicate that PBX3 at least interacts with TRIB2 and KDR to activate MAPK pathway, thereby promoting proliferation of AML cells and inhibiting their apoptosis. Collectively, a novel CD27-AS1/miR-224-5p/PBX3 axis was studied here to regulate AML cell activity through MAPK signaling pathway.

Collectively, CD27-AS1 was shown to be significantly upregulated in the AML patient samples and AML cells. Knockdown of CD27-AS1 suppressed cell proliferation and increased cell apoptosis in HL-60 and KG-1 cells. However, forced expression of CD27-AS1 showed the opposite effects. CD27-AS1 increased PBX3 expression through sponging miR-224-5p. CD27-AS1 knockdown or miR-224-5p overexpression blocked the MAPK signaling through PBX3 silencing. Therefore, the results suggested that CD27-AS1 could regulate AML cell progression through a miR-224-5p/PBX3/MAPK signaling pathway, which may provide new insights for noncoding RNA-related therapeutic intervention of AML treatment.

## Supplementary information

Supplementary Figure Legends

Supplementary Figure 1.

Supplementary Figure 2.

Supplementary Figure 3.

Supplementary Figure 4.

## Data Availability

The data that support the findings of this study are available from the corresponding author upon reasonable request.
