# Supplementary material for: LncRNA CD27-AS1 promotes acute myeloid leukemia progression through the miR-224-5p/PBX3 signaling circuit
Source: Cell Death Dis. 2021 May 18;12(6):510. doi: 10.1038/s41419-021-03767-9 (PMC8131722; doi:10.1038/s41419-021-03767-9)
Supplement: Supplementary file 1 — Supplementary Figure Legends [file 41419_2021_3767_MOESM1_ESM.docx]

Supplementary Figure Legends

Supplementary Figure 1. (a and b) After normal CD34+ cells were infected with LV-CD27-AS1 or LV-CD27-AS1-shRNAs for 72 h, cell proliferation was detected by CCK-8 assay. *P < 0.05 in LV-CD27-AS1 versus the LV-NC; *P < 0.05 and **P < 0.01 in LV-CD27-AS1-Sh1 versus the LV-NC; #P < 0.05 and ##P < 0.01 in LV-CD27-AS1-Sh2 versus the LV-NC. N=3. Data were shown as means ± SD.

Supplementary Figure 2. (a) Correlation analysis of CD27-AS-miR-224-5p target pairs in bone marrow mononuclear cells of AML patients (N=40). (b) The relationship between miR-224-5p expression and prognosis of AML patients analyzed by the Cancer Genome Atlas database using the Oncolnc software (<http://www.oncolnc.org/>). (c and d) The binding sites between CD27-AS and miR-204-5p/miR-628 were predicted by LncBase Predicted v.2 (<http://carolina.imis.athena-innovation.gr/diana_tools/web/index.php?r=lncbasev2%2Findex-predicted>)

Supplementary Figure 3. After HL-60 and KG-1 cells were co-infected with the LV-CD27-AS1-shRNA and LV-miR-224-5p for 72 h, cell proliferation was detected by CCK-8 assay (a, b). Cell apoptosis was detected using flow cytometry (c, d). N=3. Data were shown as means ± SD. *P < 0.05, **P < 0.01, ***P < 0.001.

Supplementary Figure 4. Graphical abstract. CD27-AS1 up-regulates the expression of PBX3 through sponging miR-224-5p to further activate the MAPK pathway and ultimately regulates acute myeloid leukemia progression.
