# Supplementary figures and images for: LncRNA CD27-AS1 promotes acute myeloid leukemia progression through the miR-224-5p/PBX3 signaling circuit
Source: Cell Death Dis. 2021 May 18;12(6):510. doi: 10.1038/s41419-021-03767-9 (PMC8131722; doi:10.1038/s41419-021-03767-9)

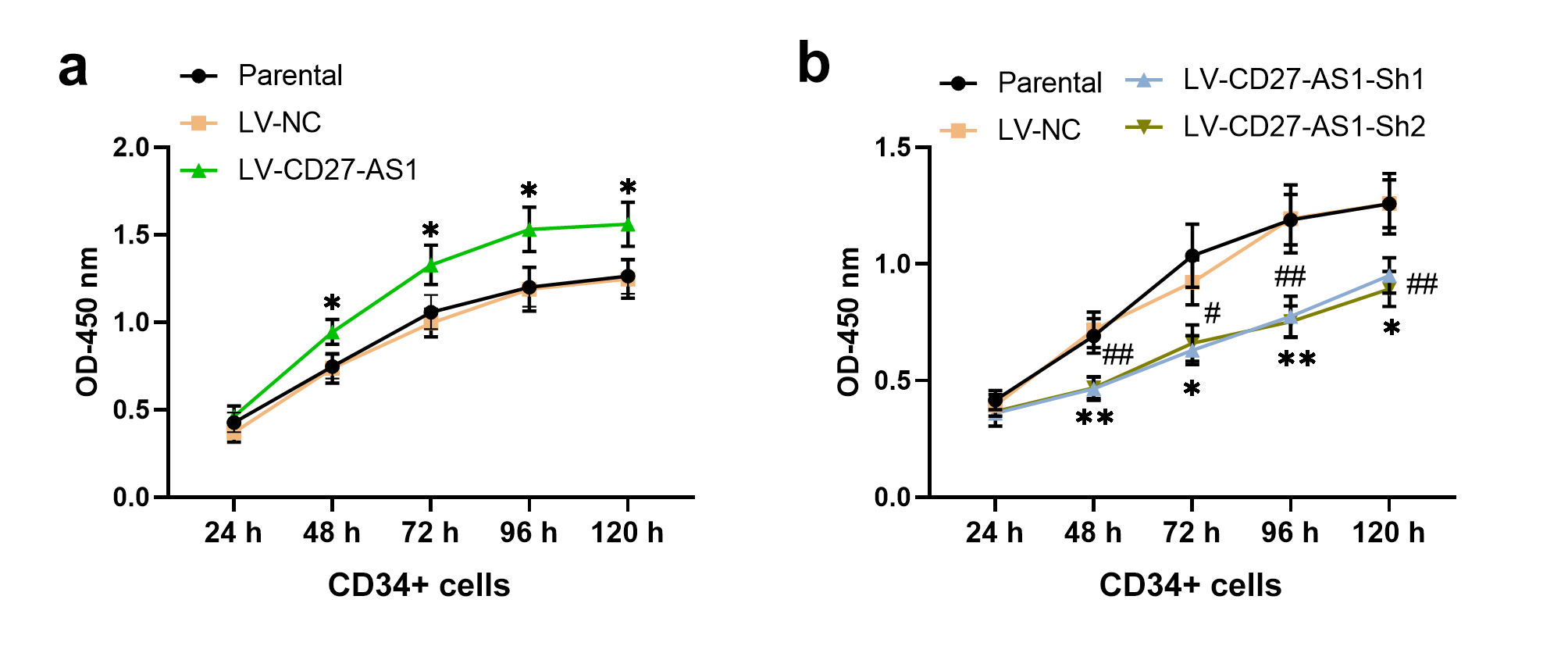

Supplement: Supplementary file 2 — Supplementary Figure 1. [file 41419_2021_3767_MOESM2_ESM.tif]

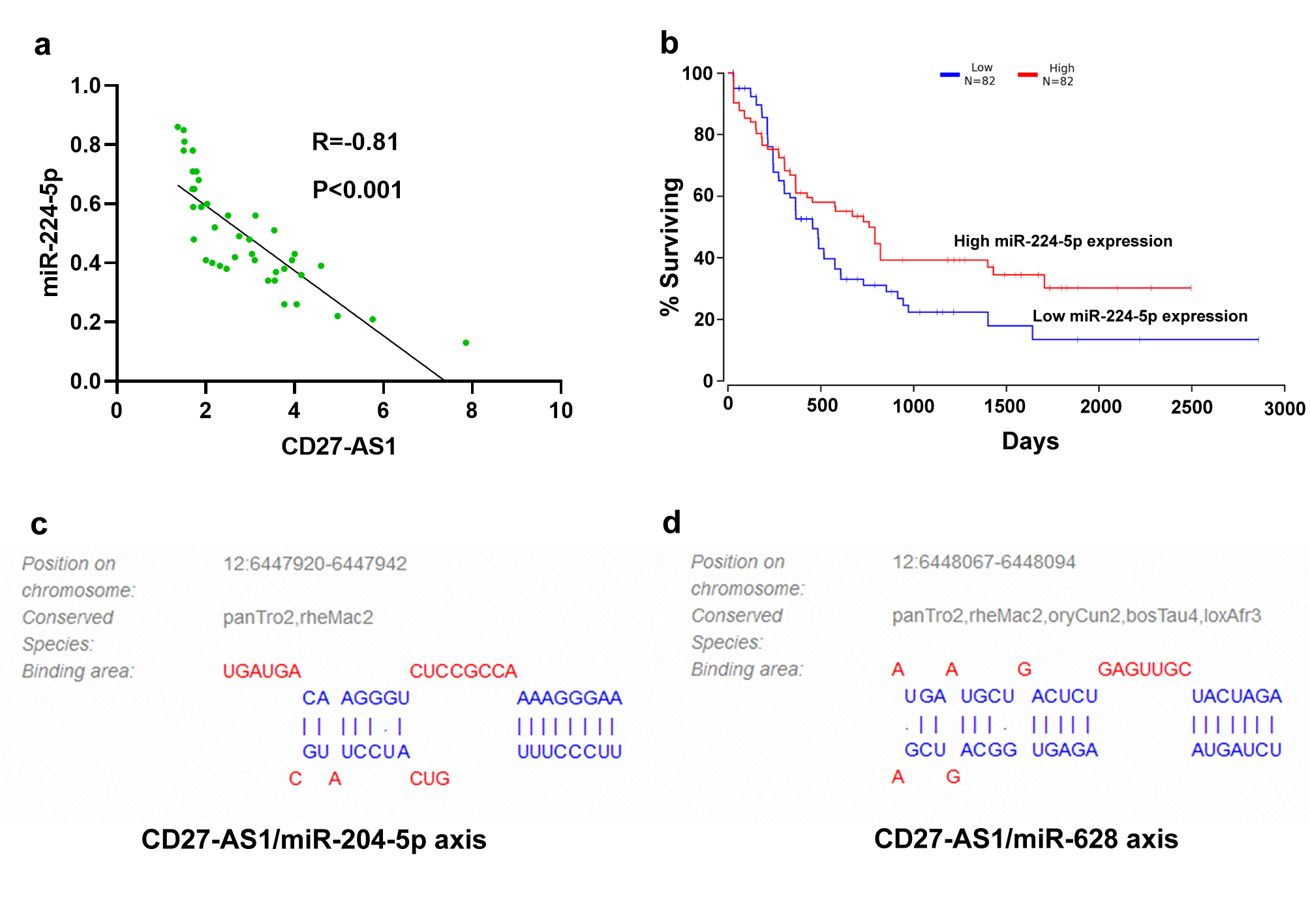

Supplement: Supplementary file 3 — Supplementary Figure 2. [file 41419_2021_3767_MOESM3_ESM.tif]

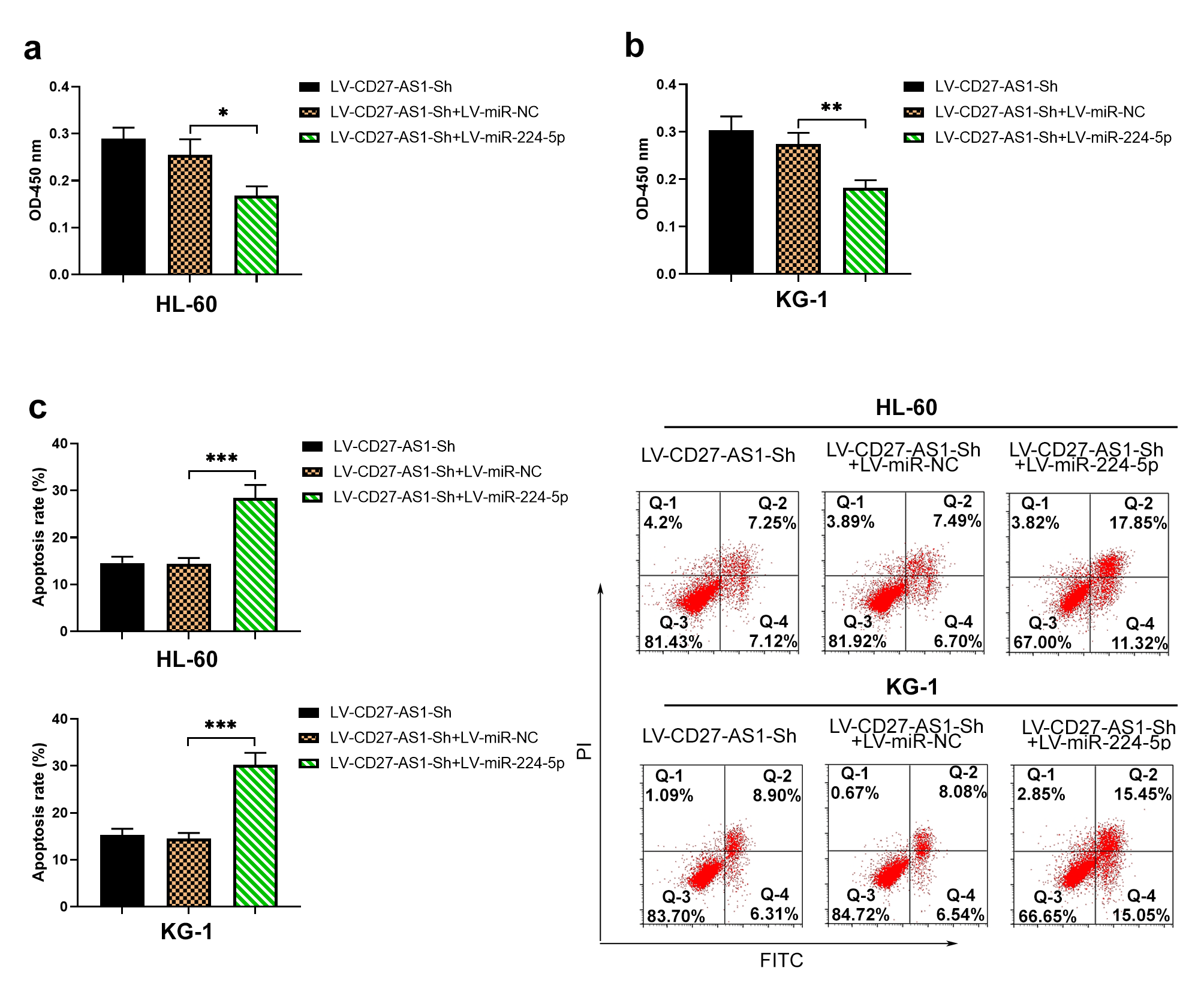

Supplement: Supplementary file 4 — Supplementary Figure 3. [file 41419_2021_3767_MOESM4_ESM.tif]

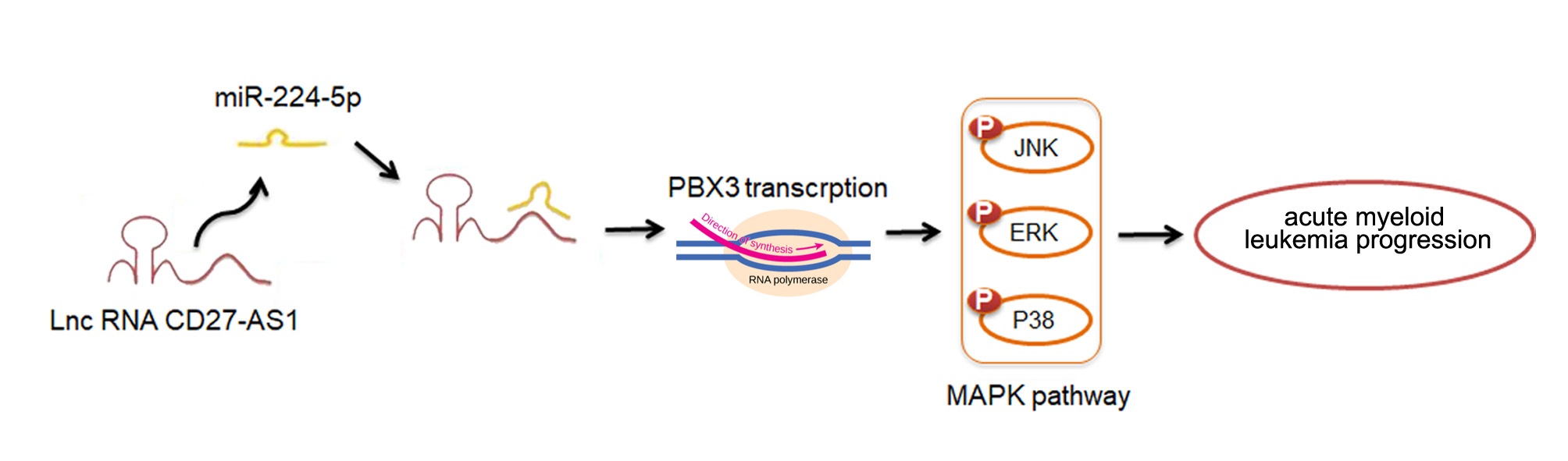

Supplement: Supplementary file 5 — Supplementary Figure 4. [file 41419_2021_3767_MOESM5_ESM.tif]
